# Hydrothermal oxidative desulfurization of thiophene to sulfate: the effect of MoO_*x*_, WO_*x*_ and carbon supports

**DOI:** 10.1039/d5se01500f

**Published:** 2026-04-30

**Authors:** Cheng Chang, Frédéric Vogel, Oliver Kröcher, David Baudouin

**Affiliations:** a PSI Center for Energy and Environmental Sciences, Paul Scherrer Institute Forschungsstrasse 111, 5232 Villigen PSI Switzerland; b Institute of Chemical Sciences and Engineering, École Polytechnique Fédérale de Lausanne, (EPFL) 1015 Lausanne Switzerland; c University of Applied Sciences Northwestern Switzerland (FHNW), Institute for Biomass and Resource Efficiency 5210 Windisch Switzerland

## Abstract

Among various forms of sulfur, some organosulfur compounds (particularly alkyl thiophenes) in biomass are rather refractory under hydrothermal conditions, posing a threat to the catalysts used in catalytic hydrothermal gasification (cHTG). In petrochemistry, alkyl thiophenes are usually treated by oxidative desulfurization (ODS) under mild conditions and removed in the form of sulfones, generating a sulfur-free product stream. ODS could be used to oxidize organosulfur compounds to sulfate, allowing efficient separation by exploiting the low salt solubility in supercritical water. To assess the viability of ODS in a cHTG process, we explored the effect of temperature and oxidant concentration (O/S ratio) on sulfate production from the ODS of thiophene. More importantly, the impact of Mo- and W-based carbon materials on the conversion of thiophene to sulfate was investigated. Our results showed a sulfate yield below 5% at temperatures ranging from 50 °C to as high as 400 °C in pressurized water. Experiments varying the oxidant-to-sulfur (O/S) ratio revealed that lower ratios (≤12) enhanced both sulfate yield and oxygen selectivity, whereas higher ratios (58 and 116) led to decreased selectivity due to excess oxidant consumption by organic matter. Carbon nanofibers (CNFs) alone increased the sulfate yield threefold (to 2.3%) at 400 °C, an effect attributed to oxygen-containing surface groups. Acid treatment of CNFs further boosted this yield to 7%. A clear correlation between surface functionalities and catalytic activity was established using FTIR and Boehm titration. Among metal oxides, Mo(iv), in the form of MoO_2_, was identified as an active phase for oxidative desulfurization (ODS), achieving a sulfate yield of 12%, while MoO_3_ and WO_3_ showed no such activity. However, metal oxide loading altered the CNF surface properties, potentially diminishing their promotional effect. These findings provide a basis for further development of MoO_2_ catalysts supported on surface-modified carbon materials, with the goal of preserving beneficial carbon surface characteristics.

## Abstract 2

Oxidative desulfurization of thiophene in pressurized water was evaluated as a pretreatment for catalytic hydrothermal gasification. Sulfate formation was enhanced by oxygen‑rich carbon surfaces and molybdenum dioxide, highlighting the roles of oxidant ratio and surface chemistry.

## Graphical abstract

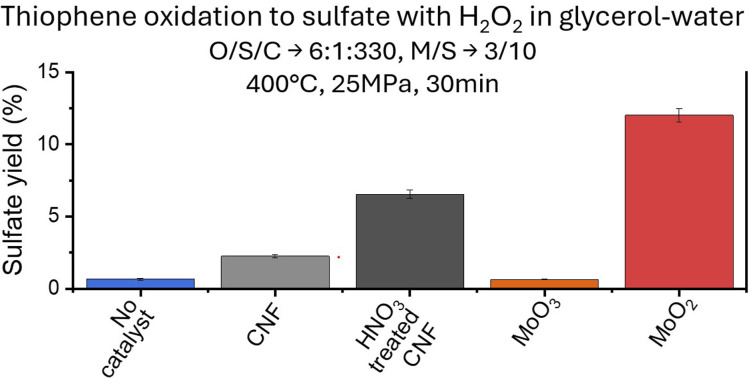

## Introduction

In the catalytic hydrothermal gasification (cHTG) process, organic matter present in a wet stream, such as sewage sludge, biogenic organic waste, or manure, can be completely converted to renewable gas, typically rich in methane. The use of supercritical water as a reaction medium allows fast kinetics, and eliminates the need to dry the feedstock prior to its valorization.^1–3^ Additionally, due to the drastic drop in dielectric constant and the derived salt solubility, SCW enables the separation of salts from the main reaction stream, facilitating their concentration into a brine phase.^4–8^ Sulfur is a prominent issue for such a process, as it is for all catalytic biomass conversion processes involving heterogeneous metal catalysts, which readily deactivate through sulfidation.^9–11^ In cHTG, hydrocarbons from biomass can transform various sulfur sources into H_2_S, which preferentially blocks the surface active sites of the catalyst.^9,12,13^ With the development of the cHTG process, inorganic sulfur species such as sulfate salts and hydrogen sulfide can be removed by salt separation^4–8^ and by chemisorption over sulfur scavengers,^11,14^ respectively. Sulfur scavengers, such as Zn-based^11,15^ or more recently Cu- and CeO_2_-based materials,^16,17^ can be used for the decomposition of a broad variety of organosulfur compounds (OSCs) such as alkyl sulfides, alkyl disulfides or thiols, and the subsequent absorption of the H_2_S formed. However, alkyl thiophenes are more recalcitrant, and their formation during cHTG of various biomass feedstocks is favored.^16,18,19^ Therefore, studying how alkyl thiophenes are formed, how to prevent their formation and how to eliminate them from the supercritical stream is crucial for lengthening the lifetime of sulfur scavengers and for protecting the catalyst, bringing economical value to the development of cHTG.

Oxidative desulfurization is a common method applied in petrochemistry to remove alkyl thiophenes from crude oil. In this process, thiophenes can be oxidized to sulfones over fixed-bed catalysts together with an oxidant.^20–23^ Subsequent separation of water-soluble sulfones by extraction or absorption allows the generation of low-sulfur oil products. Many studies on ODS were carried out with petroleum as the feedstock and sulfone or sulfate as the oxidized sulfur product.^24–31^ A wide range of catalytic materials can effectively drive oxidative conversions of thiophenic substrates under mild conditions. For H_2_O_2_, these include transition-metal oxides and titanosilicates (zeolites), *e.g.*, Ti-MWW^32^ and Ti-Beta,^33^ porous ionic liquids,^34^ and Ti-, V-, Mo-, W- and Zr-based catalysts.^35,36^ For O_2_ activation in aerobic ODS, Fe_3_N,^37^ FeWC and W oxo-carbide catalysts have shown good performance,^38,39^ while ozone-assisted oxidative desulfurization can be promoted by transition metal complexes.^40^ However, these studies had ODS performed under mild conditions (*T* ≪ 350 °C, *P* < 3 MPa) and sometimes without water, which is not compatible with cHTG applications. In addition, in a cHTG context, it is desired to oxidize the sulfur in alkyl thiophenes all the way to sulfate, which can then be efficiently removed by salt separation thanks to the low salt solubility under supercritical water conditions.^15,41–43^ Because cHTG requires pumping an aqueous organic feed at high pressure, employing a liquid oxidizer is ideal, as it ensures efficient mass transfer during preheating, whereas a gaseous oxidizer would remain as a separate phase until the water reaches its critical point, creating localized high oxidizer concentrations and an associated explosion hazard.

To facilitate ODS with H_2_O_2_ in the cHTG process, Mo- and W-based catalysts can be expected to be the most active transition metal catalysts for ODS.^44–48^ Carbon materials, such as activated carbon (AC) and carbon nanofibers (CNFs), are the supports with the best compromise between stability, performance, specific surface area, and cost in supercritical water.^49–52^ A few studies have focused on Mo- and W-based carbon material for conventional ODS,^53–56^ but none under cHTG conditions.

This paper aims to bridge the gap in ODS studies of organosulfur compounds between petrochemical research and catalytic hydrothermal gasification (cHTG), thereby exploring a potential pathway for desulfurizing cHTG product streams upstream of a salt separator. Thiophene was chosen as a recalcitrant model organosulfur compound,^16,19^ together with H_2_O_2_ as the oxidant and glycerol as an organic model compound for biomass. This paper investigates the oxidative desulfurization of thiophene to sulfate under hydrothermal conditions, with a focus on near- and supercritical water environments typically encountered in continuous hydrothermal gasification (cHTG). The focus was not on the oxidation of the hydrocarbon framework, but on the selective transformation of sulfur. Particular attention was given to the role of carbon-supported molybdenum and tungsten oxides in influencing both sulfate yield and sulfur oxidation selectivity.

## Experimental

### Materials and methods

Glycerol (>99%, Carl Roth GmbH & Co. KG) was diluted in deionized water (DI H_2_O) to 20 wt% for the batch reactor experiments. Thiophene (>99%) was purchased from Fluka and diluted to 20 mmol L^−1^ in the glycerol–water solution before reaction, representing a typical concentration of sulfur (0.064%) in wet biomass.^57^ H_2_O_2_ (30%, Merck) was diluted shortly before the test in the model solution based on the O/S ratio for each experiment. After the batch reactor experiments, isopropanol (>99.9%, Fisher Scientific AG) was used for rinsing the reactor.

The Mo- and W-based catalysts were prepared using the incipient wetness impregnation (IWI) method on two supports, activated carbon AC-CGRAN (AC) from Cabot and carbon nanofibers NC7000 (CNF) from Nanocyl, crushed and sieved to 250–500 µm. Mo and W precursor solutions were prepared by dissolving ammonium molybdenum tetrahydrate ((NH_4_)_6_Mo_7_O_24_·4H_2_O) (>99%, Fisher Scientific AG) and ammonium metatungstate hydrate ((NH_4_)_6_H_2_W_12_O_40_) (≥99.9%, Fisher Scientific AG), respectively, in DI H_2_O. The metal concentration was then determined as the desired metal loading multiplied by the mass of the catalyst, divided by the total pore volume. The support was impregnated by drop-wise addition of the Mo or W precursor solution under vigorous stirring. The impregnated support was then dried overnight in air at 120 °C before calcination for 5 h in a quartz tubular furnace heated to 500 °C (5 °C min^−1^) under argon (60 mL min^−1^). According to the literature,^58^ the corresponding ammonium salt decomposes at high temperature into metal(vi) oxides, along with ammonia and water evacuated by the flowing argon. Due to different reports^58–64^ on the oxidation state of Mo after calcination, the catalysts prepared in this way are referred to as MoO_*x*_/AC, MoO_*x*_/CNF, WO_*x*_/AC, and WO_*x*_/CNF (Table 1). To prepare HNO_3_-treated CNF, as received CNF was immersed in 65% HNO_3_ in a round bottom flask equipped with a reflux condenser and heated in a water bath at 95 °C. After 2 h, the acid treated CNF was carefully filtered and washed with DI water until the pH of the filtrate became neutral. Afterwards, the acid treated CNF was dried overnight.

**Table 1:** Metal loading and starting materials used for the catalyst synthesis

Catalysts	Metal loading (wt%)	Ammonium salt	Support
MoO_*x*_/AC	13.5	(NH_4_)_6_Mo_7_O_24_·4H_2_O	AC
MoO_*x*_/CNF	13.5	(NH_4_)_6_Mo_7_O_24_·4H_2_O	CNF
WO_*x*_/AC	21.8	(NH_4_)_6_H_2_W_12_O_40_	AC
WO_*x*_/CNF	21.8	(NH_4_)_6_H_2_W_12_O_40_	CNF

The hydrothermal experiments (Table 2) were conducted in a stainless steel batch reactor system developed in-house.^57^ The detailed experimental procedure is described in a previous study.^65^ In this study, the reaction temperature ranged from 50 °C to 400 °C. O/S ratios were mainly maintained at 0, 6 and 12, to study the effect of the O/S ratio under conditions similar to conventional ODS conditions. The O/S ratio is kept in this range to prevent overoxidation of S-free organics. The effect of the relatively high O/S ratios of 58 and 116 on the oxidation selectivity was also studied in supercritical water, for which O/C ratios were around 1/6 and 1/3, respectively. Note that such high O/C ratios will ultimately not only lead to carbon oxidation and hence CO_2_ formation, but also favor H_2_ production over CH_4_,^66^ both of which are undesirable in cHTG, where methane is typically targeted.

**Table 2:** List of experiments performed and conditions applied. For all tests, thiophene concentration and residence time were fixed at 20 mmol L^−1^ and 30 min, respectively

Experiment no.	Potential catalysts	Temperature (°C)	Glycerol (wt%)	H_2_O_2_ (mmol L^−1^)	Pressure (MPa)	O/S/C ratio^a^	Metal/S ratio^b^
1	—	400	20	60	25	6 : 1 : 330	—
2	—	350	20	60	18	6 : 1 : 330	—
3	—	300	20	60	11	6 : 1 : 330	—
4	—	250	20	60	7	6 : 1 : 330	—
5	—	200	20	60	5	6 : 1 : 330	—
6	—	150	20	60	4	6 : 1 : 330	—
7	—	100	20	60	3	6 : 1 : 330	—
8	—	50	20	60	2.5	6 : 1 : 330	—
9	—	400	0	0	25	0	—
10	—	350	0	0	18	0	—
11	—	400	20	0	25	0	—
12	—	400	20	120	25	12 : 1 : 330	—
13	—	400	20	580	25	58 : 1 : 330	—
14	—	400	20	1160	25	116 : 1 : 330	—
15	—	400	0	60	25	6 : 1 : 4	—
16	—	350	0	60	18	6 : 1 : 4	—
17	AC	400	20	60	25	6 : 1 : 330	0^c^
18	CNF	400	20	60	25	6 : 1 : 330	0^c^
19	MoO_*x*_/AC	400	20	60	25	6 : 1 : 330	3 : 10
20	MoO_*x*_/CNF	400	20	60	25	6 : 1 : 330	3 : 10
21	WO_*x*_/AC	400	20	60	25	6 : 1 : 330	3 : 10
22	WO_*x*_/CNF	400	20	60	25	6 : 1:330	3 : 10
23	HNO_3_ treated CNF	400	20	60	25	6 : 1 : 330	0
24	MoO_2_	400	20	60	25	6 : 1 : 330	3 : 10
25	MoO_3_	400	20	60	25	6 : 1 : 330	3 : 10
26	WO_3_	400	20	60	25	6 : 1 : 330	3 : 10
27	MoO_*x*_/CNF-3h	400	20	60	25	6 : 1 : 330	3 : 10

a In the O/S/C ratio, O takes only H_2_O_2_ into account and excludes the oxygen from glycerol.

b Mo or W.

c The mass of pure carbon materials added was kept the same as that of metal-loaded materials.

Each experiment was performed twice. After each experiment, the gas phase was bubbled through an alkaline trap and collected for microGC analysis (see details in ref. 65). The aqueous phase was poured out directly, after which 4 mL isopropanol (IPA) was added into the reactor to wash out the liquid residue. This organic phase is referred to as the IPA phase. The detailed experimental protocol is described in the SI. Ion chromatography (IC) was used for the determination of sulfate. UV-Vis was used for the determination of hydrogen sulfide. Gas chromatography with a sulfur chemiluminescence detector (GC-SCD) was used for the identification of volatile organosulfur compounds and to determine thiophene conversion. For the catalysts, N_2_ physisorption was used to measure the specific surface area and total pore volume. X-ray diffraction (XRD) was used to determine the crystal phase of the metal catalysts. Transmission electron microscopy (TEM) was used to assess the presence of particles on the catalyst support. Fourier transform infrared (FTIR) spectroscopy was used to qualitatively identify the surface functional groups of the carbon material, while Boehm titration was used for quantification of the oxygen-containing acidic sites on the surface.^67,68^ Details of these analytical methods are described in the SI.

## Results & discussion

Among the various organosulfur compounds that can be formed during supercritical water treatment of biomass, thiols, dialkyl disulfides and various thiophenes are the most frequent ones.^57^ Among these compounds, thiophenes are the most stable in supercritical water,^69^ due to the low electron density of their S atom. A preliminary series of ODS tests under cHTG conditions, at 400 °C and 25 MPa, was performed with various organosulfur compounds typically found in effluents produced from the hydrothermal treatment of biomass in the presence of H_2_O_2_ and glycerol (same reaction conditions as exp. 1, Table 2). The results showed that alkylthiols were the most sensitive to oxidation and thiophene the least, with the following sulfate yield order: 1-pentanethiol (29%) > benzothiophene (14%) > DMDS (9%) > thiophene (0.6%).^70^ Because of its stability towards oxidation, thiophene was selected as a model organosulfur compound to probe the efficiency of potential catalytic material in the ODS of organosulfur compounds in hydrothermal and supercritical water.

### Influence of temperature on the hydrothermal oxidative desulfurization of thiophene

**Fig. 1:**
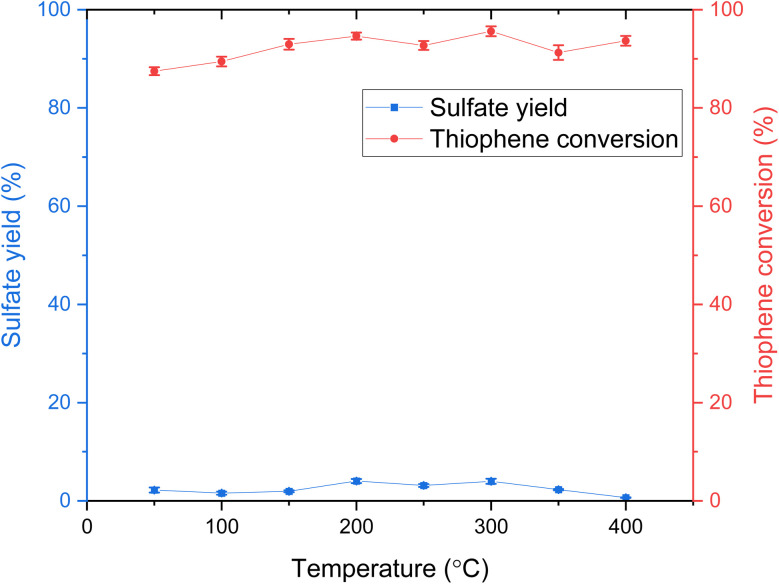
Influence of temperature on thiophene conversion and sulfate yield in ODS (results from experiments 1 to 8, with 20 wt% glycerol, 20 mmol L^−1^ thiophene, 60 mmol L^−1^ H_2_O_2_ for 30 min, O/S/C = 6 : 1 : 330, see Table 2).

The influence of temperature on thiophene conversion and sulfate yield in hydrothermal ODS was studied between 50 and 400 °C. Thiophene conversion was found to be already 87% at 50 °C. The thiophene conversion slowly increased from *ca.* 87% to 94% as the temperature rose from 50 °C to 200 °C, as shown in Fig. 1. Within the temperature range of 250 °C to 400 °C, the thiophene conversion fluctuated around 90%. The highest conversion was around 95% at 300 °C, and the lowest conversion was around 87% at 50 °C. The respective standard deviation is relatively small compared to the corresponding mean value. The results show that the effect of temperature on sulfate formation or thiophene conversion was insignificant under the reaction conditions in this study. In terms of sulfate production, the change in sulfate yield over the temperature range of 50 °C to 400 °C was found to be limited, with the yield remaining below 5%. A slightly increased yield between 200 °C and 300 °C was observed, with a maximum of around 5% in this range. The selectivity of sulfate production was very low compared to thiophene conversion.

The high conversion of thiophene, coupled with low selectivity toward sulfate, suggests that thiophene rapidly reacts to form another/other product(s) during the temperature ramp-up. These products may still contain sulfur (organosulfur compounds) or may result in sulfur being released in forms other than sulfate. This indicates that either the kinetics toward complete oxidation are slow, or the selectivity for sulfur oxidation is inherently low under the tested conditions (reductive conditions).

To assess the role of water alone on thiophene conversion and sulfate yield (blank experiment), experiments were carried out with only thiophene in water as the feedstock. Two different temperatures were chosen, 400 °C and 350 °C, for comparison between supercritical water conditions and subcritical water conditions (exp. 9 and exp. 10 in Table 2). Fig. S1 shows that thiophene conversions were around 99% and 98% at 400 °C and 350 °C, respectively, and the corresponding sulfate yields were around 0.5% and 3%, respectively. A small amount of sulfate was formed, presumably due to the oxidation of thiophene by the residual oxygen dissolved in the water of the feedstock. In this blank experiment, the concentration of hydrogen sulfide collected in the liquid trap was below the detection limit within the temperature range studied, indicating negligible H_2_S production. This result is in agreement with the data from Lu's study,^71^ which showed that thiophene in supercritical water at (450 °C) hardly decomposes to produce H_2_S (with only 0.0012% yield).

The volatile organosulfur compound (VOSC) products were below the detection limit of the GC-SCD for all experiments (0.02 ppm of S^72^), as shown in the chromatogram in Fig. S2. This result shows that no new VOSCs were produced from the reaction between thiophene, H_2_O_2_ and glycerol during all the tests performed. The fact that the conversion of thiophene was comparable from 400 °C down to 50 °C and that no VOSCs were formed under any of these conditions indicates that thiophene does not decompose under hydrothermal conditions below 400 °C, but rather forms polar or larger non-volatile compounds that still contain sulfur. The GC-SCD result in Fig. S3 shows that there were no VOSC products formed when exposed to pure water at 350 °C and 400 °C either, which is in line with the literature.^71^ This indicates that thiophene or its decomposition products are converted to non-volatile S-compounds, possibly *via* dimerisation/oligomerisation.

The fact that the experiment in the absence of H_2_O_2_ leads to high thiophene conversion cannot be explained by the presence of small amounts of oxygen in the system (O_2_dissolved_/S is *ca.* 0.013 : 1). Hydrolysis can be excluded because volatile decomposition products would have been formed and observed by GC-SCD.^73,74^ The conversion of thiophene to non-volatile thienylthiophene (dimer) or to di/tetrahydrohydroxythiophenes, catalysed by first-row transition-metal cations, was reported in pure water at 240 °C and 3.4 MPa,^75^ and might partially explain the conversion of thiophene at high temperature, assuming leaching of Ni from the stainless steel. Finally, thiophene *S*-alkylation or electrophilic reactions with the reactive carbonyl intermediates derived from glycerol dehydration, can lead to thiophene-based alkylated or condensation products.^76^ The conversion of thiophene observed even at low temperature likely originates from a reaction with glycerol, but its origin could not be clearly evidenced.

### Influence of the O/S ratio on oxidative desulfurization of thiophene

**Fig. 2:**
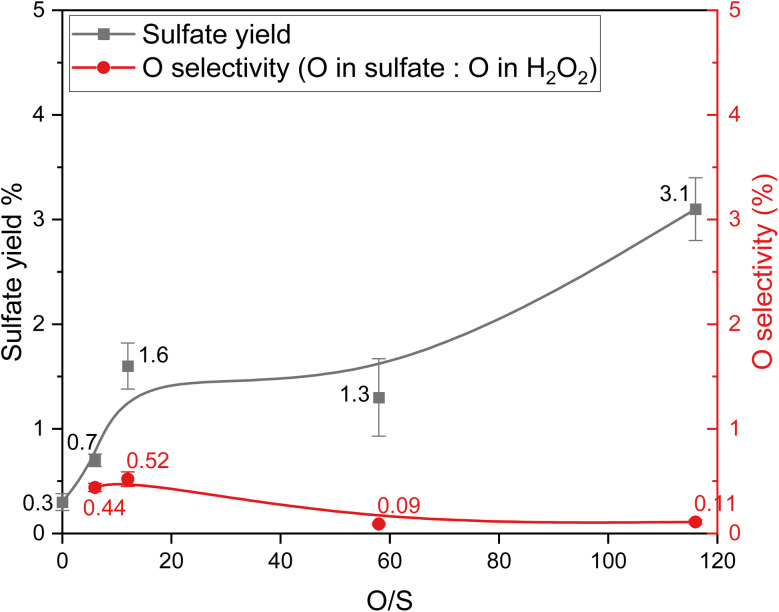
Influence of the O/S ratio on the sulfate yield and oxygen selectivity for the ODS of thiophene (results from experiments 1 and 11–14: 20 wt% glycerol, 20 mmol L^−1^ thiophene, 400 °C and 25 MPa for 30 min, S/C = 1 : 330, see Table 2).

The effect of the O/S ratio during ODS of thiophene under cHTG conditions was studied by examining sulfate production and oxygen selectivity at different O/S ratios. From Fig. 2, it can be seen that increasing the O/S ratio from 0 to 12 caused an increase in the sulfate yield from 0.3% to 1.6%. The yield remained stable when increasing the O/S ratio to 58 (within standard deviation) and then further rose to 3.1% at an O/S ratio of 116. To evaluate the overall selectivity of H_2_O_2_ toward sulfate formation, the oxygen selectivity was analyzed, defined as the ratio of oxygen incorporated into sulfate to the total oxygen amount supplied by H_2_O_2_ (see eqn (S3)). The oxygen selectivity behaved differently from the sulfate yield. Initially, the increase in oxygen selectivity was insignificant when the O/S ratio increased from 6 to 12. Then, the oxygen selectivity decreased to 0.09% as the O/S ratio increased to 58. At this point, adding more H_2_O_2_ to increase the O/S ratio did not significantly affect the oxygen selectivity, which reached 0.11% when the O/S ratio was 112.

These results reveal that increasing the O/S ratio in the region ≤ 12 is beneficial for sulfate production and oxygen selectivity, but further increasing the O/S ratio to 58 has an adverse impact on oxygen selectivity. Doubling the O/S ratio to 116 would double the sulfate yield, but the oxygen selectivity remained the same. The low selectivity might be a result of the highly reactive conditions met in (near) supercritical water, which involve the formation of small reductants such as H_2_, short alcohols or aldehydes,^77^ prone to oxidation. Another possibility is that ODS took place during the temperature ramp-up and the thiophene oxidation product was reduced back once the SCW conditions in the presence of glycerol were reached. It is unclear how fast H_2_O_2_ reacts with thiophene and glycerol (and their decomposition products) in the experimental setting of this study. When the reaction system was overloaded with the oxidant (O/S ≥ 12), sulfate production continued to increase; however, excess oxidant – including unselective ˙OH radicals from H_2_O_2_ – was likely consumed by organic matter in the feedstock or might even reduce the already formed sulfate. This leads to a decrease in oxygen selectivity, suggesting that while thiophene conversion was rapid, the reaction pathway favors alternative products over selective sulfur oxidation. Within the 30 min residence time, further increasing the H_2_O_2_ concentration of the feed did not increase the oxygen selectivity for sulfate production from the ODS of thiophene.

Additionally, Fig. S4 shows that increasing the H_2_O_2_ concentration can lead to the measurable production of volatile organosulfur compounds, which are considered as byproducts in the process of sulfate production. Based on these results, an O/S ratio of 6 (H_2_O_2_ concentration at 60 mmol L^−1^) was considered optimal and chosen as the reaction conditions for subsequent experiments. Different feed compositions showed that the addition of 20 mmol L^−1^ thiophene and 60 mmol L^−1^ H_2_O_2_ did not affect the gas produced from the non-catalytic gasification of glycerol (Fig. S5). However, glycerol itself could affect the selectivity of the ODS of thiophene to sulfate, as its decomposition products in SCW include reducing gases such as CO and H_2_. Since glycerol was fixed at 20 wt%, 60 mmol L^−1^ H_2_O_2_ resulted in a low O/C ratio of 1/55, which might have hindered the oxidative desulfurization (ODS) of thiophene to sulfate.

To probe whether the sulfate production from thiophene oxidation was hindered by the low O/C ratio caused by glycerol, experiments were carried out without the addition of glycerol (with a high O/C ratio of 1.5) in the feed for comparison. Two different temperatures were chosen, 400 °C and 350 °C, for the comparison between supercritical water conditions and subcritical water conditions (exp. 15 and exp. 16 in Table 2).

Fig. S6 shows that thiophene conversion in the experiments without glycerol was around 99% at both 400 °C and 350 °C, slightly higher than in the cases with glycerol, which was around 92%. This can be explained by the addition of glycerol in the feedstock, which competes with thiophene in reacting with H_2_O_2_, thus leading to a lower thiophene conversion. This explanation is supported by the sulfate yield results in Fig. S7, in which the sulfate yields from the experiments without glycerol were around 15% and 33% at 400 °C and 350 °C, respectively, much higher than in the cases with glycerol, which were around 0.7% and 2%, respectively. Note that at the O/S/C ratio of 6 : 1 : 4 used, a maximum of 46% of the full oxidation of thiophene to CO_2_, H_2_SO_4_ and H_2_O can be reached. According to literature, the formation of sulfoxide (C_4_H_4_SO) or sulfones (C_4_H_4_SO_2_) during ODS is preferred. Thiophene sulfone is the most stable and common product before ring cleavage, indicating that the conditions applied favor the formation of sulfate. The higher sulfate yield observed at 350 °C compared to 400 °C may be attributed to increased reduction of intermediate organosulfur compounds by thiophene decomposition products, *e.g.* CO, at the higher temperature, which competes with sulfate formation and limits its accumulation. It should also be noted that VOSC products were below the detection limit without glycerol, meaning that decomposition of thiophene to volatile organosulfur side products did not occur under these conditions, or these compounds were readily oxidized to non-volatile compounds. This would be explained by the faster oxidation kinetics of, *e.g.*, thiols or disulfides that can form upon thiophene decomposition,^70^ resulting in thiophene being the dominating organosulfur compound observed. These results underline the low selectivity of H_2_O_2_ oxidation of thiophene under these conditions.

The low sulfate selectivity further indicates that several competing pathways operate under these reducing conditions. Because thermochemical sulfate reduction is extremely slow^78^ and the S(+vi) → S(+iv) step is rate-limiting,^79^ sulfate formed in this system is unlikely to be further reduced. Thus, the limited sulfate yield may arise from partially oxidized sulfur intermediates being diverted into non-volatile organosulfur compounds, and/or from unselective H_2_O_2_ reactions with other organic species present.

The strong suppression of sulfate formation by glycerol can be attributed to intense competition between thiophene and the large excess of sulfur-free organic species (glycerol and its decomposition products), present at an S/C_glycerol_ molar ratio of 1 : 326. Under the applied conditions—low viscosity, complete miscibility of reactants, and high temperature and pressure—H_2_O_2_ is expected to react rapidly and unselectively with any available organic compound. This large excess of reductants relative to H_2_O_2_ promotes highly unselective oxidation pathways, thereby limiting sulfur oxidation to sulfate. Achieving high sulfate selectivity therefore requires a catalyst capable of steering the reaction toward selective sulfur oxidation and enhancing the corresponding reaction kinetics.

### Influence of Mo- and W-based carbon materials on ODS of thiophene

As described in the former section, the use of catalysts to accelerate thiophene oxidation towards sulfate is necessary. To prevent excessive oxidation of organics, which is undesired in the catalytic hydrothermal gasification to methane, an O/C ratio of 1/55 was kept for the subsequent experiments.

**Fig. 3:**
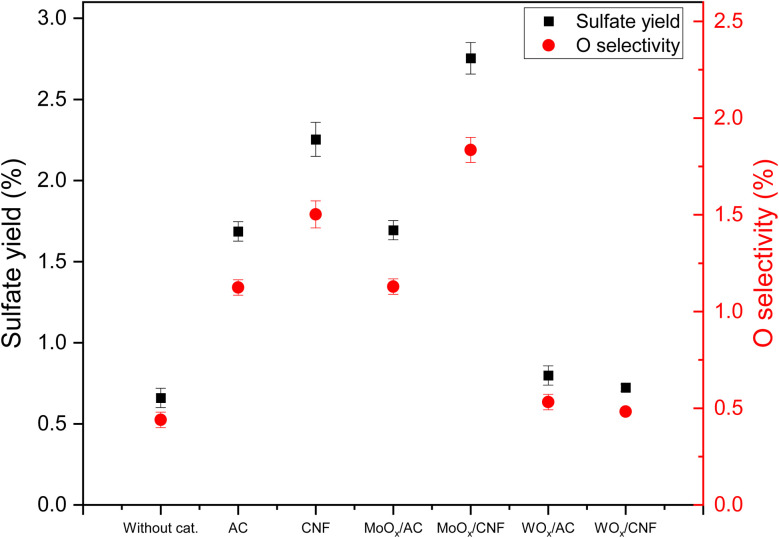
Influence of various Mo- and W-based carbon materials on the sulfate yield and oxygen selectivity from the ODS of thiophene (results from experiments 1 and 17–22: 20 wt% glycerol, 20 mmol L^−1^ thiophene, 60 mmol L^−1^ H_2_O_2_, 25 MPa pressure and 30 min residence time, see Table 2).

Fig. 3 shows the influence of various Mo- and W-based carbon supported materials, as well as the carbon supports themselves, on the sulfate yield and oxygen selectivity in the ODS of thiophene. First, it can be observed that AC and CNF supports increased the sulfate yield by a factor of 2.4 and 3.4 when compared to the test without the addition of any material. The oxygen selectivity followed the same trend, with approximately 1.4% over AC and 1.8% over CNF, which is a 3 to 4-fold increase compared to the material-free test. The positive effect of the carbon materials might result from the oxygen-containing surface functional groups on the supports.

After loading these two carbon supports with WO_*x*_, the sulfate yield over WO_*x*_/AC and WO_*x*_/CNF both dropped to around 0.7%, which is nearly the same as in the case without material addition. Their oxygen selectivity followed the same trend. However, loading MoO_*x*_ on the carbon supports exhibited a different result. The sulfate yield over MoO_*x*_/CNF increased to 2.8%, which is 20% higher than that over pure CNF, while the sulfate yield over MoO_*x*_/AC was the same as over pure AC (1.7%). There are a few hypotheses that could explain these results: 1. WO_*x*_ might favor competing reactions, preventing sulfate formation; 2. WO_*x*_ might favor the reduction of sulfate or ODS intermediates 3. WO_*x*_ altered the surface functionalities of the supports favoring thiophene oxidation to sulfate. In parallel, MoO_*x*_ seems to participate in sulfate formation, or in changing the nature of the surface of the carbon support, *e.g.* by the formation of more carbon surface functionalities through C surface oxidation. It can, however, not be excluded at this stage that MoO_*x*_ decreases the surface functional groups of the carbon support active in sulfate formation, while MoO_*x*_ contributes to the ODS reaction. The difference between MoO_*x*_/AC and MoO_*x*_/CNF could be explained by the presence of different forms or phases of MoO_*x*_, or the much higher mesoporous surface area of CNF (AC being mostly microporous, see Table S3 and Fig. S13) and hence the intensified possible effect of MoO_*x*_ on surface carbon active sites.

Note that experiments were conducted to quantify the sulfate adsorption capacity of AC, CNF, MoO_*x*_/AC and MoO_*x*_/CNF (blank adsorption tests, see the SI). These materials were found to adsorb sulfate at a level corresponding to 0.19%, 0.02%, 0.12%, and 0.015% sulfate yield (see Table S1), which are negligible compared to the values obtained during the hydrothermal ODS tests.

To better understand the effect of the carbon surface on the sulfate yield, FTIR spectra of the fresh catalysts were measured. The FTIR spectra of CNF, MoO_*x*_/CNF and WO_*x*_/CNF are presented in Fig. S8. Due to the strong absorption of carbon, it had to be highly diluted in KBr to allow exploitable IR transmission, at the expense of the signal to noise ratio. Yet, absorption bands at around 1260 cm^−1^, 1540 cm^−1^, and 1585 cm^−1^ were observed on CNFs, which can be assigned to the C–O bond vibration in lactone,^80^ the asymmetric stretching vibration of the carboxylate group (–CO_2_^−^),^81^ and the C=C stretching mode associated with nanofiber surface defects,^82^ respectively. After loading MoO_*x*_ and WO_*x*_, the absorption at 1260 cm^−1^ (C–O vibration in lactone) clearly decreased in the order CNF > MoO_*x*_/CNF > WO_*x*_/CNF while that at the other two bands varied slightly. This supports the previous assumption that WO_*x*_ and MoO_*x*_ altered the surface properties of the CNF.

**Fig. 4:**
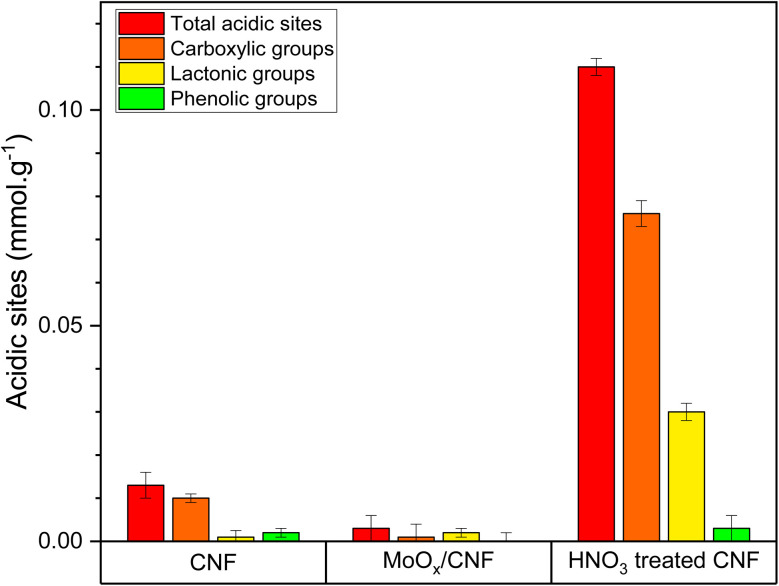
The type and concentration of acidic functional groups on different materials: as received (untreated) CNF, on HNO_3_-treated CNF and on MoO_*x*_ supported on (untreated) CNF.

Boehm titration was used to quantify the acidic sites on the potential catalysts, which allowed a more direct analysis of the oxygen-containing surface functional groups. With bases of various strengths (NaHCO_3_, Na_2_CO_3_, and NaOH), different acidic oxygen-containing groups are neutralized distinctively. Fig. 4 reveals that after the loading of MoO_*x*_, the total amount of acidic sites decreased, with only lactonic groups as the main contributor, represented by the C–O bond vibration in lactone at 1260 cm^−1^ on the FTIR spectrum of MoO_*x*_/CNF. The decrease in oxygen-containing surface functional groups on MoO_*x*_/CNF further supports the previous assumption that the loading of MoO_*x*_ could alter the surface properties and cover active sites, which is in agreement with the FTIR results.

**Fig. 5:**
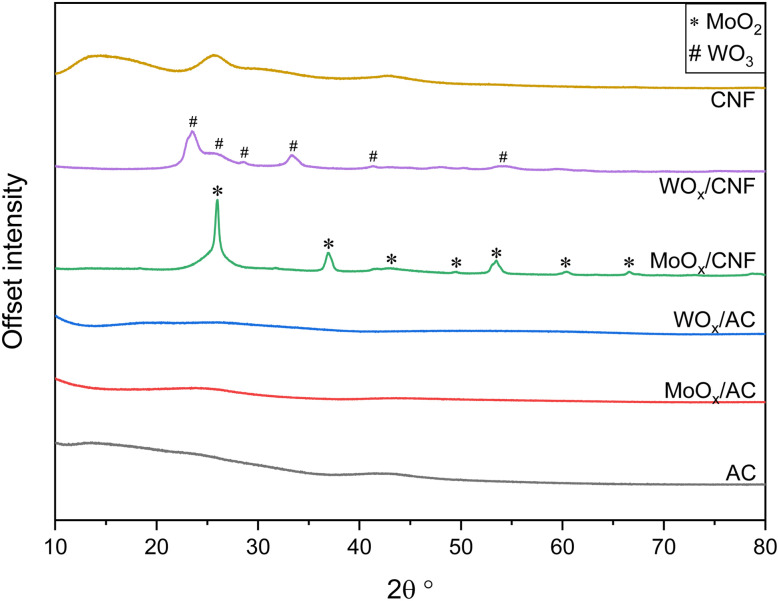
XRD patterns of fresh Mo- and W-based catalysts and the neat supports AC and CNF.

XRD characterization was carried out on fresh AC, CNF, MoO_*x*_/AC, MoO_*x*_/CNF, WO_*x*_/AC, and WO_*x*_/CNF to evaluate the crystalline phases, and indirectly the oxidation state of Mo and W present in the supported metal oxides. The XRD patterns are shown in Fig. 5. The diffraction reflections (“diffraction peaks”) on the XRD pattern of MoO_*x*_/CNF correspond to the characteristic peaks of MoO_2_. No signals of MoO_3_ were found for MoO_*x*_/CNF. The diffraction peaks observed for WO_*x*_/CNF are characteristic of WO_3_ and no other type of tungsten phase was found. These results indicate that the oxidation state of Mo was reduced from +6 (ammonium salt used for the synthesis) to +4 after the thermal treatment performed in an inert atmosphere, while that of W remained the same at +6. The only explanation is that the CNF support acted as a reductant for MoO_3_ during the thermal treatment, reducing it to MoO_2_. In the same group of the periodic table, W has a higher atomic number (74) than Mo (42), resulting in a greater effective nuclear charge. The standard reduction potentials of MoO_3_/MoO_2_ and WO_3_/WO_2_ indicate a higher reducibility of molybdenum oxide (0.25 V *vs.* 0.036 V, respectively). Note that oxycarbides of W or Mo normally form only under high-temperature, non-aqueous, carbon-rich conditions (typically ≥600–900 °C),^83,84^ while thermodynamics show that neither metal should be reduced below +IV between 150–550 °C in pure or reductive supercritical water.^85^ Therefore, forming W or Mo oxycarbides under the conditions used here is extremely unlikely.

In the case of MoO_*x*_/AC and WO_*x*_/AC, no diffraction peaks could be observed. This suggests that the metal oxides on AC are amorphous or smaller than *ca.* 5 nm. TEM was used to further assess the presence of particles, but hardly any particles could be observed (see Fig. S9 and S10).

Apart from the effect of surface functional groups, it has also been reported that the formation of Mo species with an oxidation state lower than +6 from MoO_3_ leads to high activity for the ODS of organosulfur compounds with Mo(v) as the most active species.^86^ The combination of XRD results and measured sulfate yields suggest that loading MoO_*x*_ onto CNF promoted the sulfate yield from the ODS of thiophene due to the presence of Mo(iv). The difference between AC and CNF supported MoO_*x*_ can be explained either by highly dispersed MoO_*x*_, as evidenced by XRD and TEM, which altered the carbon surface functionalities, compensating the promoting effect of the Mo(iv) or by MoO_*x*_, which is only present in the micropores of the AC, resulting in very low accessibility of the metal.

Note that Mo- and W-based phases present in the fresh catalyst are prone to evolve during the tests under reductive supercritical water.

### The effect of oxygen-containing surface functional groups

**Fig. 6:**
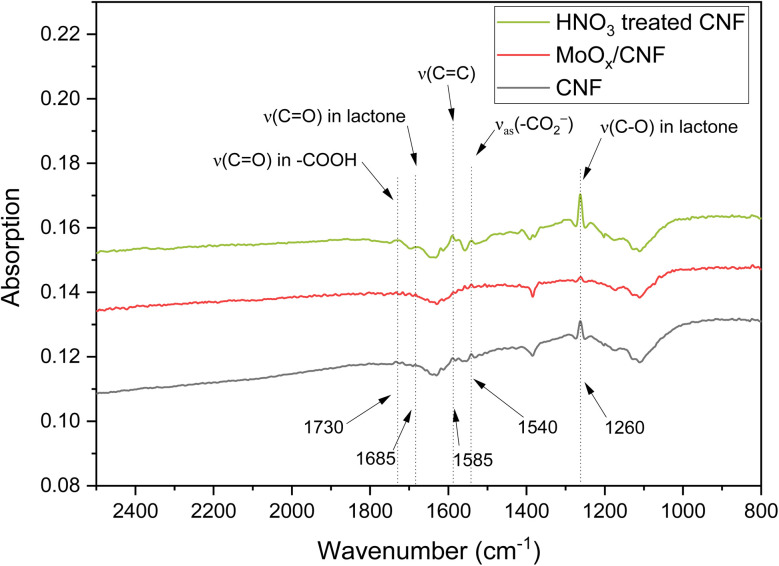
FTIR results of the modified CNF materials (diluted 500-fold with KBr; the CNF used to prepare MoO_*x*_/CNF was not treated with HNO_3_).

The potentially promoting effect of oxygen-containing surface functional groups on the sulfate yield in the ODS of thiophene was studied by treating CNF with HNO_3_ (reflux for 2 h at 95 °C), which is known to oxidize the carbon surface.^87–89^ FTIR spectroscopy shows an increase in the absorption bands at around 1260 cm^−1^, 1540 cm^−1^, and 1585 cm^−1^ after the HNO_3_ treatment, which correspond to the C–O vibration in lactone, the asymmetric stretching vibration of the carboxylate group, and the C=C stretching vibration of CNF surface defects, respectively (see Fig. 6). Additionally, two new bands appeared in the spectrum of the HNO_3_-treated CNF at around 1685 cm^−1^ and 1730 cm^−1^, which can be assigned to the C=O bond vibration in lactonic groups and carboxylic groups, respectively.^90–92^ This confirms that the HNO_3_ treatment of CNF produced more oxygen-containing surface functional groups on CNF.

Boehm titration (Fig. 4) of the HNO_3_-treated sample shows that the oxidation treatment of CNF led to a considerable increase in the concentration of acidic sites, which is around 8 times higher than that of the untreated CNF.

**Fig. 7:**
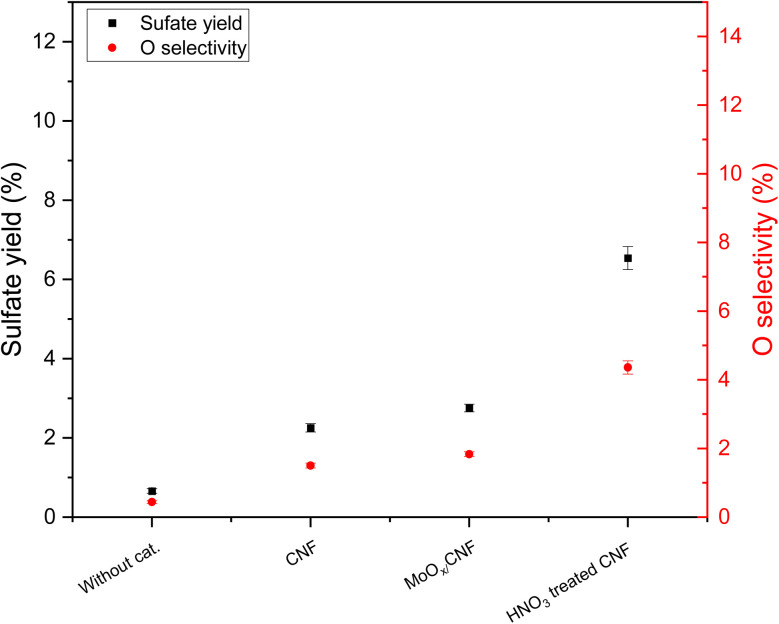
Influence of HNO_3_ treatment of CNF on sulfate yield and oxygen selectivity (results from experiments 1, 18, 20 and 23).

The same reaction conditions were applied in the batch reactor experiment (exp. 23 in Table 2) using HNO_3_-treated CNF as potential catalyst for the ODS of thiophene. Fig. 7 shows that the sulfate yield increased significantly to almost 8% over HNO_3_-treated CNF compared to around 2.3% over the original CNF, and the oxygen selectivity increased from below 0.5% to above 4%. Note that the quantity of acidic sites on the surface of CNF and HNO_3_-treated CNF represents approximately 3 and 9% of the sulfate formed, considering conservatively that four acidic sites are needed to oxidize one thiophene to sulfate, and they therefore cannot account for a stoichiometric (non-catalytic) oxidation of thiophene to sulfate. It can be inferred that the addition of oxygen-containing surface functional groups on the CNF promoted the sulfate yield from the ODS of thiophene. This result supports our previous hypothesis regarding the catalytic effect of oxygen-containing surface functional groups on selective thiophene oxidation towards sulfate.

**Fig. 8:**
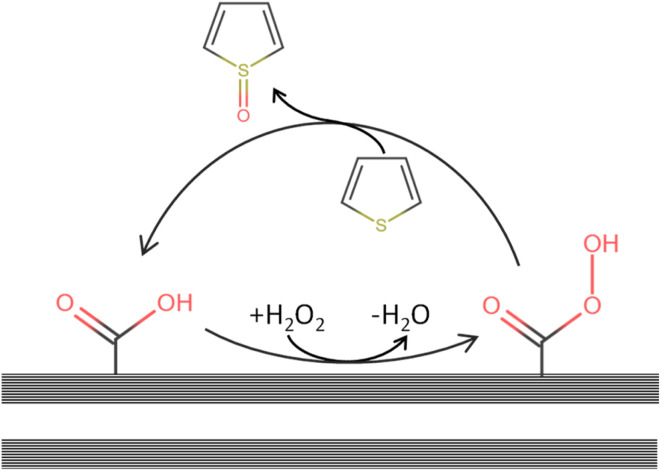
Reaction pathway involving the formation of a percarboxylicacid-type active site leading to the oxidation of thiophene to, here, its sulfoxide.

It has been reported that organosulfur compounds (*e.g.* thiophene or dibenzothiophene) can adsorb onto surface sites^93^ of the carbon catalyst, and our results, together with prior studies, suggest that activated carbon likely enhances ODS by generating surface-bound percarboxylic acid species formed through the reaction of carboxylic acids with H_2_O_2_,^94–96^ as depicted in Fig. 8. Thiophene is expected to have π–π interactions with the CNF surface, favouring its reaction with percarboxylic acid. This proposed percarboxylic-acid-type active site aligns with established reaction pathways for liquid-phase oxidation of thiophenic sulfur compounds, where percarboxylic acids produced from carboxylic acids (typically formic or acetic acid) and hydrogen peroxide drive the oxidation process.^21,97,98^ Moreover, *ex situ* formation of surface-anchored percarboxylic acids on mesoporous silica has been demonstrated to effectively oxidize BT and DBT model compounds.^99^

Carbon nanofiber and activated carbon (AC) has long been recognized as one of the more hydrothermally stable support materials for heterogeneous catalysts (at 23–30 MPa, 380–450 °C), particularly in comparison to silica, alumina, and zeolites.^52,100^ However, the stability of activated carbon is not unconditional, and its surface functional groups are subject to transformation under supercritical water conditions. However, to the best of our knowledge, the stability of such functionalities at the surface of supercritical water stable carbon materials has not been systematically studied, making the long-term stability of functionalised CNF difficult to assess.

Supporting MoO_*x*_ on CNF led to a decrease in the total acidic sites, likely as a result of the adsorption of Mo on these sites. It is noteworthy that despite MoO_*x*_/CNF containing four times fewer oxygen-containing surface groups than CNF, it still produces a slightly higher sulfate yield. It is assumed that the addition of Mo(iv) can also promote the sulfate yield from the ODS of thiophene. The promoting effect of Mo(iv) addition might be weaker than the adverse effect of surface functional group removal on MoO_*x*_/CNF; thus, the sulfate yield over MoO_*x*_/CNF was still higher than that over pure CNF.

### The effect of Mo(iv)

**Fig. 9:**
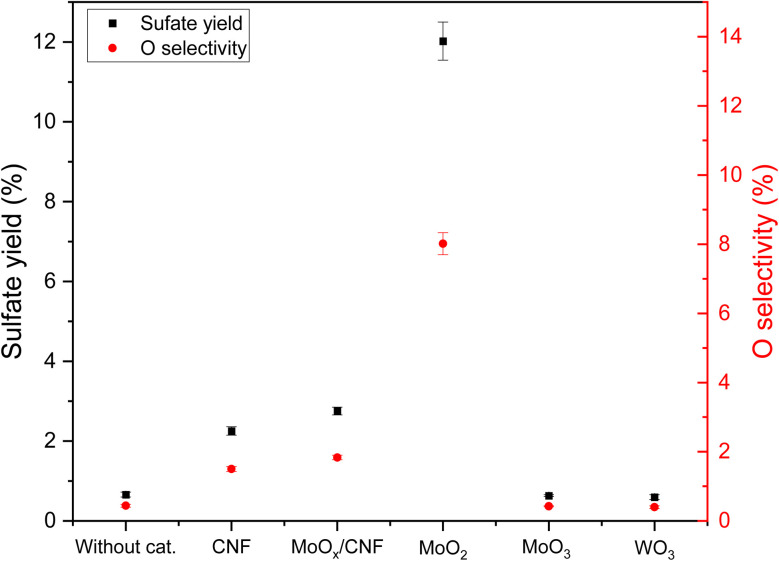
The effect of pure MoO_2_, MoO_3_, and WO_3_ on sulfate yield and oxygen selectivity (results from experiments 1, 18, 20, and 24 to 26).

Batch reactor experiments (exp. 24 to exp. 26 in Table 2) were carried out using pure MoO_2_, MoO_3_ and WO_3_ as potential catalysts to further study the assumed promoting effect of Mo(iv) on the sulfate yield in the ODS of thiophene. To allow for a comparison, the same molar quantity of active metal as in the supported metal oxide materials was used. Note that the specific surface areas of the carbon-supported materials are two orders of magnitude larger than those of the pure oxides (see Table S2), but the surface areas of MoO_3_ and MoO_2_ are similar. The surface area of the oxide (active metal) is not known in MoO_*x*_/CNF, but the dispersion (the proportion of metal at the surface, and hence the proportion of metal that is active) is expected to be much higher than in MoO_3_ and MoO_2_ considering the TEM observations made. Fig. 9 shows that the sulfate yield over MoO_2_ increased to around 12%, while that over MoO_3_ and WO_3_ and without catalysts was around 0.6%. The oxygen selectivity followed the same trend and reached approximately 8%. This result is in line with the assumption that Mo(iv) promotes sulfate production, while Mo(vi) and W(vi) do not have any impact.

Because the metal/S ratio (3 : 10) was kept constant and because the dispersion on MoO_*x*_/CNF is much higher than in MoO_2_, this result points out that the selectivity towards sulfate is much higher with MoO_2_ than with MoO_*x*_/CNF. Sintering or coking alone can therefore not explain the low performance of highly dispersed MoO_*x*_/CNF compared to poorly dispersed MoO_2_. Oxidation of Mo to Mo(vi) or rapid leaching of highly dispersed Mo species from MoO_*x*_/CNF may account for the reduced performance. Although these effects could not be assessed due to the small catalyst mass and the presence of coke, recent work showed substantial leaching of highly dispersed NiMo catalysts during heating from room temperature to 400 °C in pure water.^56^ While the conditions here differ and are less prone to leaching, Mo leaching during the batch experiments cannot be excluded, particularly on the highly dispersed MoO_*x*_/CNF (Fig. 9).

Then, the effect of calcination temperature of the catalysts on the sulfate yield was investigated. It has been reported that MoO_3_ can be reduced to MoO_2_ on the surface of carbon materials at high temperatures due to the reductive nature of carbon.^101^ At 500 °C, an increasing amount of MoO_2_ was formed with the increase in residence time during the reaction between MoO_3_ and carbon. The original calcination time used for the preparation of MoO_*x*_/CNF was 5 h. By shortening the calcination time to 3 h (exp. 27 in Table 2), less MoO_2_ formation was expected. The XRD patterns (Fig. S11) of both catalysts show little difference between both, except for smaller and broader diffraction peaks, indicative of lower MoO_2_ formation, which is in agreement with Zhang's observations.^101^

Fig. S12 shows that the sulfate yield over MoO_*x*_/CNF-3h is around 1.5%, which is lower than that over MoO_*x*_/CNF and CNF alone, indicating that the promoting effect of Mo(iv) on the sulfate yield is weaker with shorter calcination time, and inadequate loading of Mo(iv) (or calcination of CNF) might not be able to compensate for the drop in catalytic activity due to the alteration of surface functional groups. This result also supports that Mo(iv) is a key factor ins enhancing the sulfate yield.

Molybdenum is well known for its redox capability in oxidative processes such as ODS;^102^ however, under the predominantly reductive conditions applied here, thermodynamic modeling indicates that MoO_2_ is the dominant stable phase,^85^ and any redox cycling would more likely occur between Mo(0) and Mo(iv) sites. The reduction of MoO_3_ to MoO_2_ in pressurized pure water has been experimentally observed at temperatures as low as 320 °C,^103^ consistent with thermodynamic calculations,^85^ when accounting for the measured^103^ oxygen fugacity in pure water. However, the very low activity of MoO_3_ suggests that this reduction proceeds slowly. Given that reaction conditions and medium composition evolve continuously during testing, and therefore oxygen fugacity also changes, obtaining direct experimental evidence for molybdenum-based redox mechanisms is particularly challenging and lies beyond the scope of this work.

## Conclusion

In the ODS of thiophene under cHTG-relevant conditions, temperature has little effect on conversion and does not significantly promote sulfate formation. In contrast, the O/S ratio strongly influences both sulfate yield and oxygen selectivity. A moderate increase in the O/S ratio enhances sulfate yield, but beyond a certain threshold, selectivity toward sulfate declines. This is likely due to competing oxidation reactions involving hydrocarbons in the feedstock, which consume the excess oxidant. Integrating the ODS of thiophene into a SCWG process in order to convert organosulfur compounds to sulfate would therefore negatively impact methane production.

Selectivity towards sulfate formation from thiophene during ODS reaches a maximum around 350 °C before dropping above the critical point of water. This is likely due to competitive reduction reactions in SCW caused by the rapid decomposition of organics and higher concentrations of small reductive compounds (H_2_, CO, light alcohols, and alkenes). However, the sulfate yield without the use of a catalyst remains marginal, *i.e.*, below 5%.

Catalyst screening revealed that carbon nanofiber enhances sulfate production, whereas activated carbon does not, which suggests that micropores are less relevant, probably because they become filled with coke. A clear correlation was observed between oxygen-containing surface groups and sulfate yield. By increasing CNF surface functionalities or by using pure MoO_2_, a significant increase in thiophene conversion to sulfate can be achieved.

## Conflicts of interest

There are no conflicts to declare.

## Supplementary Material

SE-010-D5SE01500F-s001

## Data Availability

The data supporting this article have been included as part of the supplementary information (SI). Supplementary information: experimental procedures, analytical methods, data analysis equations, additional characterization results (XRD, TEM, FTIR, physisorption, Boehm titration), chromatograms, control experiments, and supporting figures and tables. See DOI: https://doi.org/10.1039/d5se01500f.
