# Supplementary material for: Hydrothermal oxidative desulfurization of thiophene to sulfate: the effect of MoOx, WOx and carbon supports
Source: Sustain Energy Fuels. 2026 Apr 30;10(10):2563–74. doi: 10.1039/d5se01500f (PMC13130181; doi:10.1039/d5se01500f)

Supplementary Information (SI) for Sustainable Energy & Fuels.
This journal is © The Royal Society of Chemistry 2026

Hydrothermal oxidative desulfurization of thiophene to sulfate: effect of MoO_x, WO_x and carbon support

Cheng Chang[1,2], Frédéric Vogel[1,3], Oliver Kröcher[1,2], David Baudouin[1*]

[1] Paul Scherrer Institute (PSI), Energy and Environment, CH-5232 Villigen PSI, Switzerland

[2] Institute of Chemical Sciences and Engineering, École Polytechnique Fédérale de Lausanne, (EPFL), 1015 Lausanne, Switzerland

[3] University of Applied Sciences Northwestern Switzerland (FHNW), Institute for Biomass and Resource Efficiency, 5210 Windisch, Switzerland

Supplementary Information

General procedure for experiments

Firstly, the sand bath temperature was stabilized for 1 h once it reached the setting temperature of the experiment. Meanwhile, model solution (20 wt% glycerol with 20 mM thiophene and 60 mM H_2O_2) were loaded into the reactor with optional addition of potential catalysts. The volume of solution was maintained the same at 6.7 mL throughout this work. Mo- or W-based materials were added at a metal to sulfate molar ratio of 3:10 (and the weight of pure carbon materials addition were kept the same as the weight of metal oxide loaded materials). Then the batch reactor was sealed and connected to the system, leak tested, and purged five times with argon to ensure oxygen-free conditions, before setting the pressure to 2.5 MPa with Ar. After the batch reactor was placed into the sand bath, the residence time started counting once 350 °C was reached (around 3 minutes) when the internal environment is near-supercritical (if the target temperature is higher than the critical point). When the target residence time was elapsed, the batch reactor was quenched by immersing it in a large tank of room temperature water. After cooling down for 15 min, the pressure was released through the sampling line. Gaseous H2S in the product was trapped with an alkaline liquid trap (0.5 M NaOH) for further UV-Vis analysis, while other gas products were collected in a multi-layer foil gas sampling bag (No. 22950 from Restek) for gas chromatography analysis. The aqueous phase was poured directly into a 15 mL borosilicate glass sampling vial (G075G-27/047-H) for IC analysis, after which 4 mL isopropanol (IPA) was added into the reactor to wash out the organic phase deposited on the walls of the reactor for GC-SCD. These samples are referred to as aqueous phase and IPA phase, respectively, throughout this work. Finally, the solid residue, including the solid material added as well as potentially formed coke, was collected with a spatula.

Analytical methods

N_2 physisorption

The specific surface area of the catalysts was measured by N_2 physisorption on an Autosorb iQ-XR. Prior to the analysis, the samples were outgassed under vacuum for 3 h at 250 °C and pressure change test were set at 6.7 Pa/min every 15 min to make sure that it is fully outgassed. The specific surface area (SSA) was calculated according to the multi-point Brunauer-Emmett-Teller (BET) model in the relative pressure (P/P_0) range 0.05 – 0.3. The total pore volume was calculated at a relative pressure $P/P_0 \geq 0.99$.

X-ray diffraction

The X-ray diffraction (XRD) patterns of the catalysts were measured on a Bruker Advance D8 diffractometer with Cu-Kα radiation source (λ= 1.5406 Å) in the 20 – 80° 2θ range and a 0.03° step scan. Samples were ground in an agate mortar before being placed on the holder.

Transmission electron microscopy

High-resolution TEM images were acquired on a probe-corrected JEOL JEM-ARM200F (NeoARM) microscope equipped with a cold-field emission gun operated at 200 keV and a Gatan OneView camera. The instrument could be operated in TEM or STEM modes. Fresh materials and solid residues from the batch reactor experiments were dried over night at 125 °C and then dispersed in ethanol before being loaded on lacey carbon supported copper grids (Ted Pella Inc.).

Fourier transform infrared spectroscopy

Fourier transform infrared (FTIR) spectroscopy was often used for the characterization of surface functional groups of the carbon material[105]. The spectra of catalyst were recorded using Bruker Vertex 80 V FTIR spectrometer equipped with RT-DLaTGS detector and a sample holder for transmission measurements on pellets. Spectra were recorded in the range 400-4000 cm^{-1} with a resolution of 2 cm^{-1}. To prepare pellets, the samples were first ground to powder in an agate mortar and then mixed with KBr at a mass ratio of 1:500 to account for the strong IR absorptivity of carbon materials. A hydraulic press was used to press the resulting mixtures to discs of 10 mm in diameter at 10 MPa for 5 min. Prior to preparing the pellets, the samples and the KBr were dried overnight in a convection oven set to 120 °C. To

minimize the uptake of moisture, prepared pellets were stored in desiccator before measurements. The reported spectra are the average of 32 scans recorded relative to a background measured on a pure KBr pellet.

Ion chromatography

The sulfate concentration of the aqueous phase was quantified by ion chromatography (IC) at ambient temperature with a Metrosep A Supp 10 - 100/4.0 anion column and a guard column. Samples were diluted and filtered with a 0.22 µm PTFE hydrophilic filter before analysis. The eluent consisted of 4.0 $mmol\cdot L^{-1}$ sodium bicarbonate, 6.0 $mmol\cdot L^{-1}$ sodium carbonate and 5 $\mu mol\cdot L^{-1}$ sodium perchlorate in purified water from a Milli-Q Advantage (Millipore) unit. The flow rate was 1 $mL\cdot min^{-1}$ and the pressure 12 MPa. Recording of the data from the Metrohm 732 IC conductivity detector and analysis of chromatograms were performed with Metrohm Metrodata software. The influence of glycerol on the sulfate calibration curve of the IC analysis indicated a negligible effect of 20 wt% glycerol compared to pure water. The sulfate conversion (X_S) from TSR was calculated with Equation S1 based on the measured sulfate concentration in the feed ($C_{sulfate}$), the volume of feed solution (V_{feed}), the sulfate concentration after TSR batch reactor experiments ($C_{sulfate\_TSR}$) and the volume of aqueous phase (V_{aq}). It should be noted that V_{aq} was quantified by measuring the liquid volume directly after pouring out from the reactor without adding isopropanol. Due to experimental loss, V_{aq} is not equal to V_{feed}. A recovery test, in which model solution was introduced into the batch reactor without hydrothermal treatment and then poured out, showed that around 13 % of sulfate loss could be attributed to non-recovered liquid volume (V_{loss}). The recovery test was repeated three times and the standard deviation was 1.5 %. This experimental loss was added to the sulfate conversion in all figures of this study. The sulfate yield (Y_S) from ODS was calculated with Equation S2 based on the measured OSC concentration in the feed (C_{OSC_feed}), the stoichiometric coefficient of sulfate ($\nu_{sulfate}$) and OSC (ν_{OSC}), the volume of feed solution (V_{feed}), the sulfate concentration after ODS batch reactor experiments ($C_{sulfate\_ODS}$) and the volume of aqueous phase (V_{aq}). Oxygen selectivity (S_O) from ODS was calculated with Equation S3 based on the measured sulfate concentration ($C_{sulfate}$) and the fixed H_2O_2 concentration ($C_{H_2O_2}$).

$$XS(\%) = \frac{Csulfate \times Vfeed - Csulfate\_TSR \times (Vaq + Vloss)}{Csulfate \times Vfeed} \times 100\% \qquad S1$$

$$YS(\%) = \frac{\nu OSC \times Csulfate\_ODS \times (Vaq + Vloss)}{\nu sulfate \times COSC\_feed \times Vfeed} \times 100\% \qquad S2$$

$$SO(\%) = \frac{4 \times CODS\_after \times (Vaq + Vloss)}{2 \times CH2O2 \times Vfeed} \times 100\% \qquad S3$$

UV-vis spectroscopy

The hydrogen sulfide collected in the liquid trap was analyzed by UV-vis on a spectrophotometer from MACHEREY-NAGEL (The NANOCOLOR UV/VIS II) using methylene blue method. 10 g N,N-dimethyl-p-phenylenediamine was dissolved in 5 mL 95 % H_2SO_4 and distilled water to a total volume of 10 mL (amine-sulfuric acid stock solution). 0.25 mL amine-sulfuric acid stock solution was taken with 9.75 mL 50% H_2SO_4 and stored in a dark glass bottle (amine-sulfuric acid reagent). 100 g $FeCl_3 \cdot 6H_2O$ was dissolved in 40 mL distilled water (ferric chloride solution). 400 g $(NH_4)_2HPO_4$ was dissolved in 800 mL distilled water (diammonium hydrogen phosphate solution). With every 7.5 mL sample, 0.5 mL amine-sulfuric acid reagent and 0.15 mL ferric chloride solution were added. After 3 min, 1.6 mL diammonium hydrogen phosphate solution was also added. After another 3 min, the solution was poured into the cuvette for UV-vis analysis at 664 nm wavelength. It should be noted that H_2S in the liquid trap from all experiments in this paper was below detection limit (1.8 µM).

Gas chromatography - sulfur chemiluminescence detector

The volatile organosulfur compounds (VOSC) recovered in the IPA phase (boiling point less than 230 °C) were analyzed by gas-chromatography coupled to a sulfur chemiluminescence detector (GC-SCD, Agilent 7890A; Agilent 355) equipped with an Agilent J&W DB-Sulphur SCD column (Part No. G3903-63002; 40 m × 0.32 mm ID) using helium as the carrier gas. The SCD has a linear and equimolar response (using dibenzothiophene as internal standard) to only sulfur-containing compounds without interference from most sample matrices. The total VOSC amount (n_{VOSC}) was calculated using the equimolar method with Equation S4 based on

the sum of peak area (A_{sum}), the peak area of the internal standard dibenzothiophene (A_{DBT}), the sulfur concentration of the internal standard (C_{std_S}= 6.7 mg/L), the measured volume of the IPA phase (V_{IPA}), and the molar mass of sulfur (M_S = 32.064 g/mol).

$$nVOSC = \frac{Cstd\_S \times (Asum - ADBT)}{ADBT \times MS} \times VIPA \qquad S4$$

The OSC conversion (X_{OSC}) was calculated with Equation S5 based on the measured OSC concentration in the feed (C_{OSC_feed}), the volume of feed solution (V_{feed}), the OSC concentration after batch reactor experiments in aqueous phase (C_{OSC_aq}) and in IPA phase (C_{OSC_IPA}), the volume of aqueous phase (V_{aq}) and IPA phase (V_{IPA}).

$$XOSC(\%) = \frac{COSC\_feed \times Vfeed - COSC\_aq \times (Vaq + Vloss) - COSC\_IPA \times (VIPA + Vloss)}{COSC\_feed \times Vfeed} \times 100 \qquad S5$$

Boehm titration

By using Boehm titration and the standardization method suggested by Goertzen et al., the surface functional groups were quantified[68,69]. The occurrence of oxygen surface groups with varying acidities that may be neutralized by bases with varying strengths is the basis for this approach. In this work, $NaHCO_3$, Na_2CO_3, and NaOH were employed. The weakest base, $NaHCO_3$, exclusively neutralizes carboxylic groups; lactonic/carboxylic groups are neutralized by Na_2CO_3, and phenolic/lactonic/carboxylic groups are neutralized by NaOH. By calculating the difference, the number of moles of each surface functional group was approximated. 0.1 g carbon support (dried before weighing) was added to 25 mL of one of the three bases: 0.01 M $NaHCO_3$; 0.01 M Na_2CO_3 and 0.01 M NaOH. The samples were sealed with parafilm and stirred for 24 h at room temperature. After filtration with 0.22 µm PTFE filter, 10 mL from the sample solution was taken and neutralized by an excess of 0.01 M HCl and then titrated with 0.01 M NaOH. Back titration was stopped, and the endpoint was determined by phenolphthalein (color indicator). The amount of acid sites n_{as} are quantified by Equation S8:

$$nas = \frac{\nu HCl}{\nu B} CBVB - (CHClVHCl - CNaOHVNaOH)\frac{VB}{VA} \qquad S8$$

C_B and V_B are the concentration and volume of the base solution used for carbon support neutralization. C_{HCl} and V_{HCL} are the concentration and volume of the acid (HCl) added to the filtrate. V_A is the volume of the filtrate taken. C_{NaOH} and V_{NaOH} are the concentration and volume of NaOH that was used in the back-titration for the neutralization of the excessive acid. ν_B and ν_{HCl} are the stoichiometric coefficients of the base and HCl.

It is interesting to note that, on mesoporous graphitic carbons, it was recently proved that phenolic is the main acid site in gas-phase chemistries and unexpectedly carboxylic groups are much less acidic than phenolic groups in the graphitized mesoporous carbon due to electron density delocalization induced by the aromatic rings of graphitic carbon[106].

Sulfate blank adsorption tests

A stock solution with a concentration of 1000 mg/L sulfate was prepared by dissolving potassium sulfate (K_2SO_4) in deionized water. All sulfate solutions used in adsorption experiments were diluted from this stock solution. A certain weight (0.02 g) of the adsorbent was added into a 200 mL conical flask containing 50 mL 10 mM sulfate solution. The flasks were placed on a magnetic stirring plate at 300 rpm and room temperature. After remaining in contact for 24 h, the solid adsorbent and solution were separated by filtration, and the filtrate was collected to determine sulfate concentration. The sulfate ion concentrations in aqueous solution were measured using ion chromatography method. The detection limit of the ion chromatography method is 0.13 mg/L and the relative standard deviation is 1.25 %. All adsorption experiment were carried out in duplicate. The difference in sulfate concentration between the pre-experiment solution and the post-experiment solution would be used to calculate the total amount of adsorbed sulfate, which then divided by the weight of adsorbent is the sulfate adsorption capacity of the catalysts.

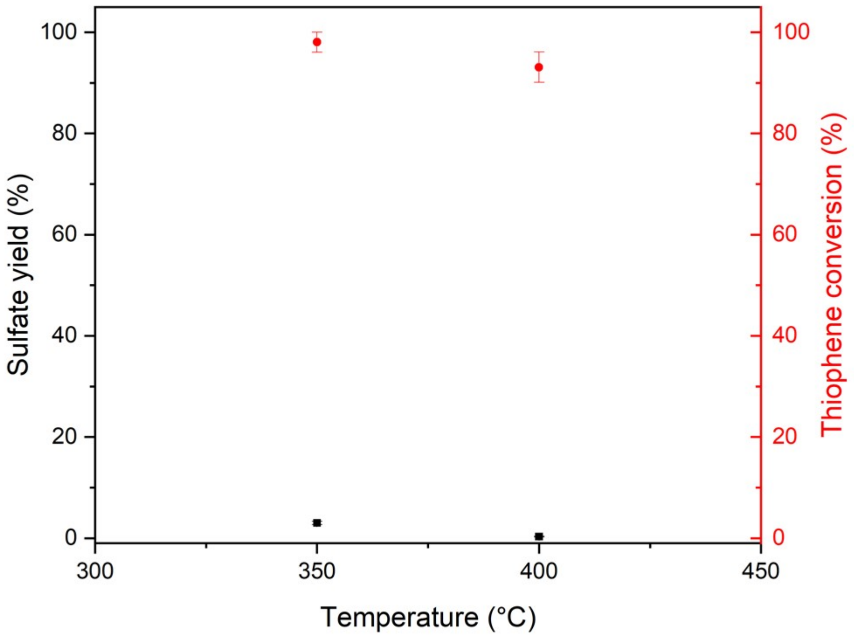

Figure S1 Sulfate yield and thiophene conversion from experiment 9 and 10 (0 wt% glycerol, 20 mM thiophene, 0 mM H_2O_2 for 30 min).

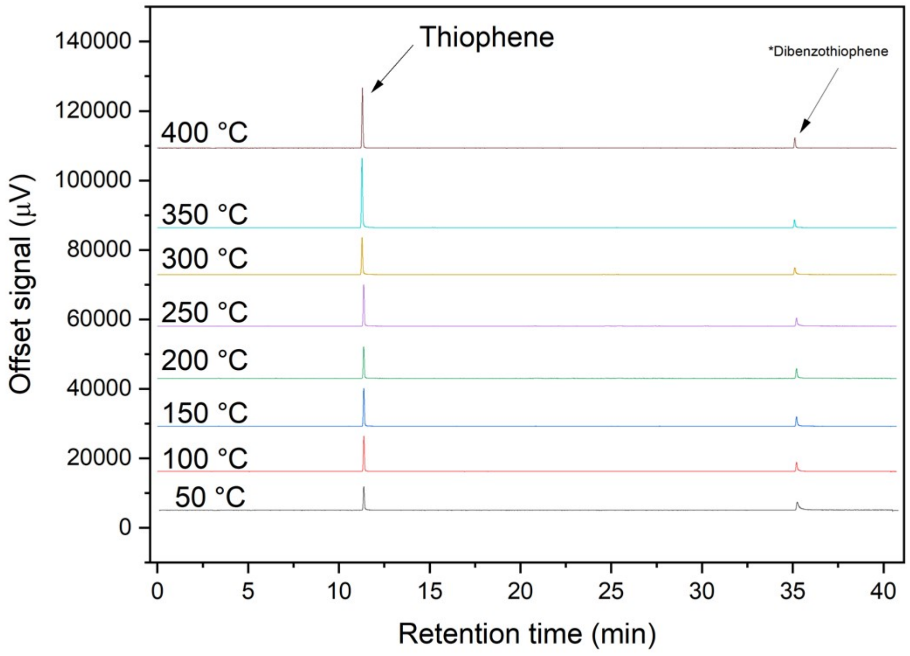

*Figure S2 GC-SCD chromatogram of the liquid product (IPA phase) from hydrothermal experiments at different temperature (*Dibenzothiophene as internal standard) (Results from experiment 1 to 8 with 20 wt% glycerol, 20 mmol·L^{-1} thiophene and 60 mmol·L^{-1} H_2O_2 for 30 min, O/S/C=6:1:330)*

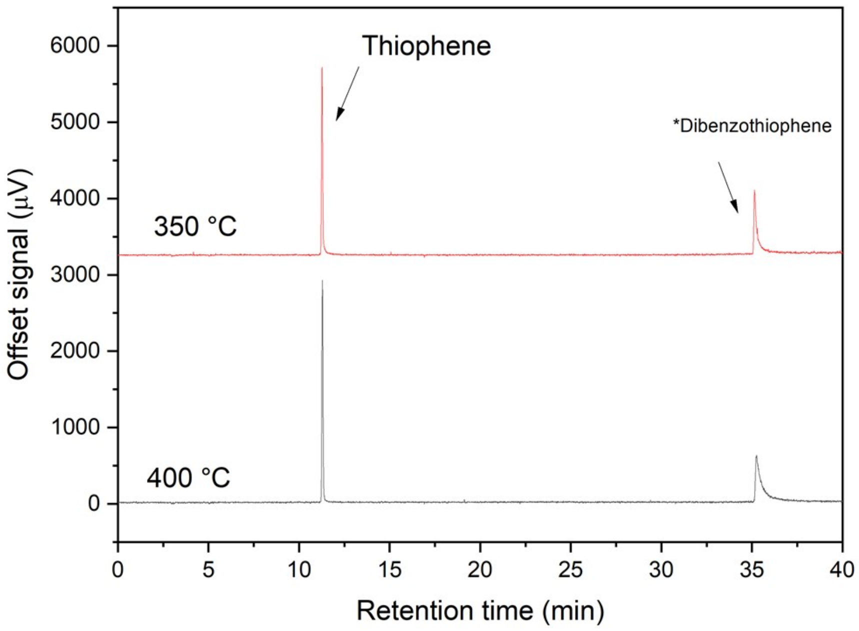

Figure S3 GC-SCD chromatogram of liquid products (IPA) from experiment 9 and 10 (0 wt% glycerol, 20 mmol·L^{-1} thiophene, 0 mmol·L^{-1} H_2O_2 for 30 min).

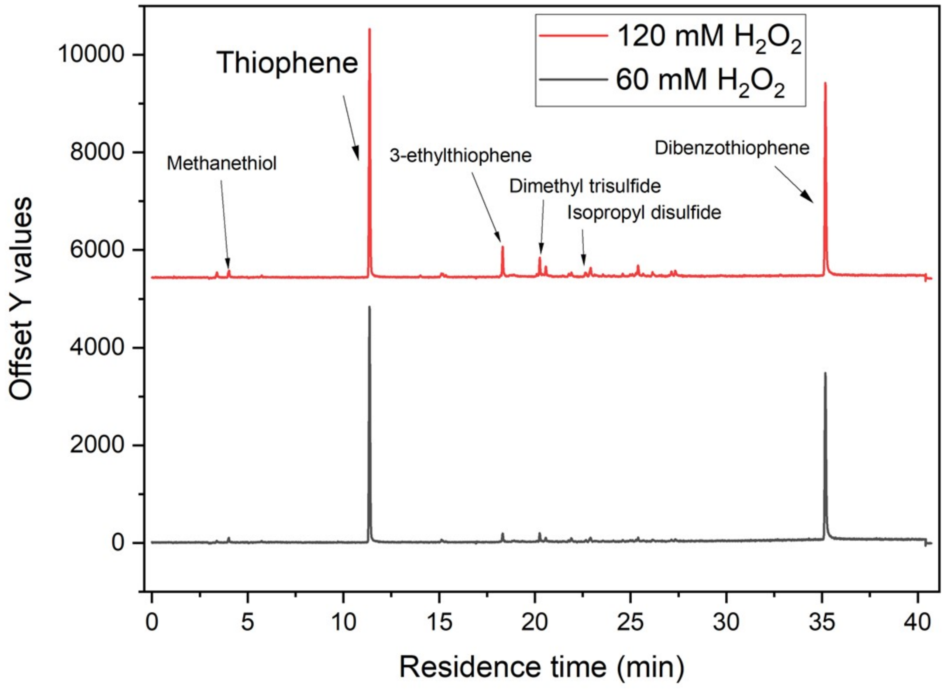

Figure S4 GC-SCD chromatogram of liquid products (IPA phase) from hydrothermal experiments with different H_2O_2 concentration. (20 wt% glycerol, 20 mmol·L^{-1} thiophene, 400 °C and 25 MPa for 30 min, O/S/C ratio 6:1:330 and 12:1:330) (results from experiment 1 and 12).

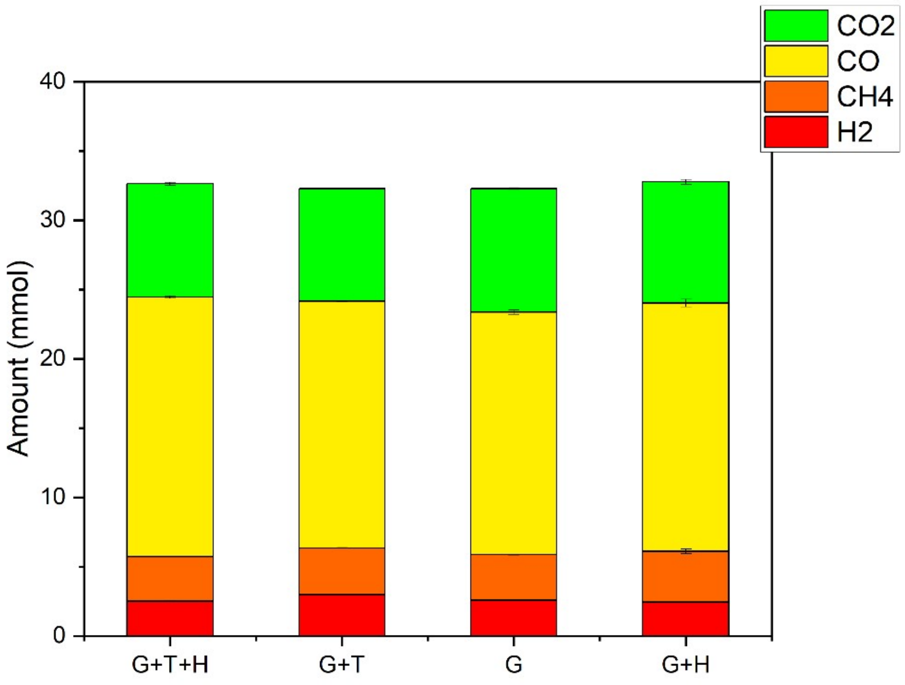

Figure S5 Gas products distribution from controlled experiments at 400 °C and 25 MPa for 30 min (G+T+H: 20 wt% glycerol, 20 mmol·L^{-1} thiophene, 60 mmol·L^{-1} H_2O_2; G+T: 20 wt% glycerol, 20 mmol·L^{-1} thiophene, 0 mmol·L^{-1} H_2O_2; G: 20 wt% glycerol, 0 mmol·L^{-1} thiophene, 0 mmol·L^{-1} H_2O_2; G+H: 20 wt% glycerol, 0 mmol·L^{-1} thiophene, 60 mmol·L^{-1} H_2O_2).

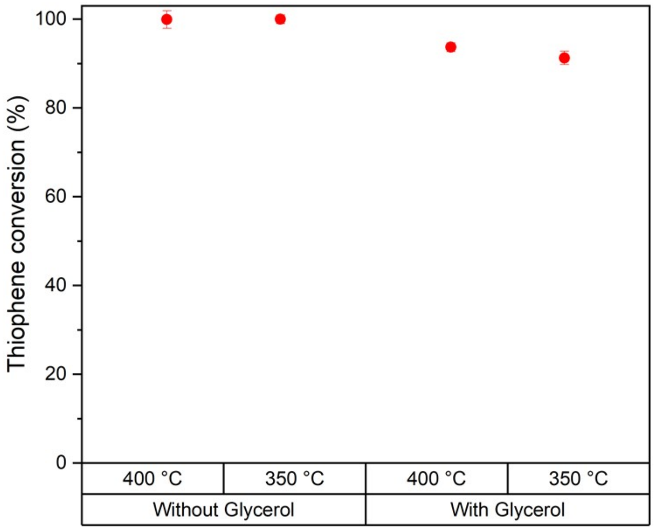

Figure S6 Thiophene conversion from experiments 1, 2, 15 and 16 (with and without glycerol at two different temperature points).

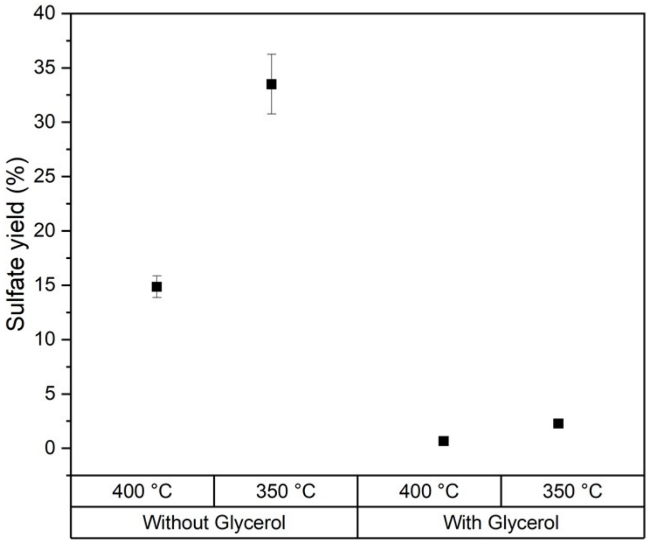

Figure S7 Sulfate yield from experiments 1, 2, 15 and 16 (with and without glycerol at two different temperature points).

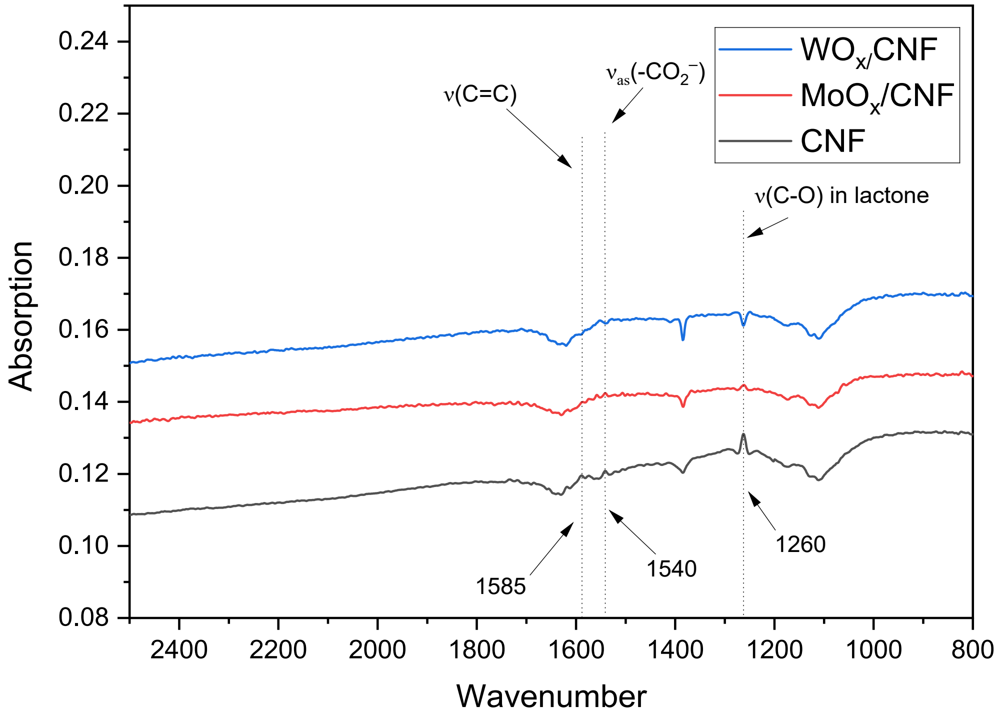

Figure S8. FTIR results from three different fresh CNF materials (diluted 500-fold with KBr).

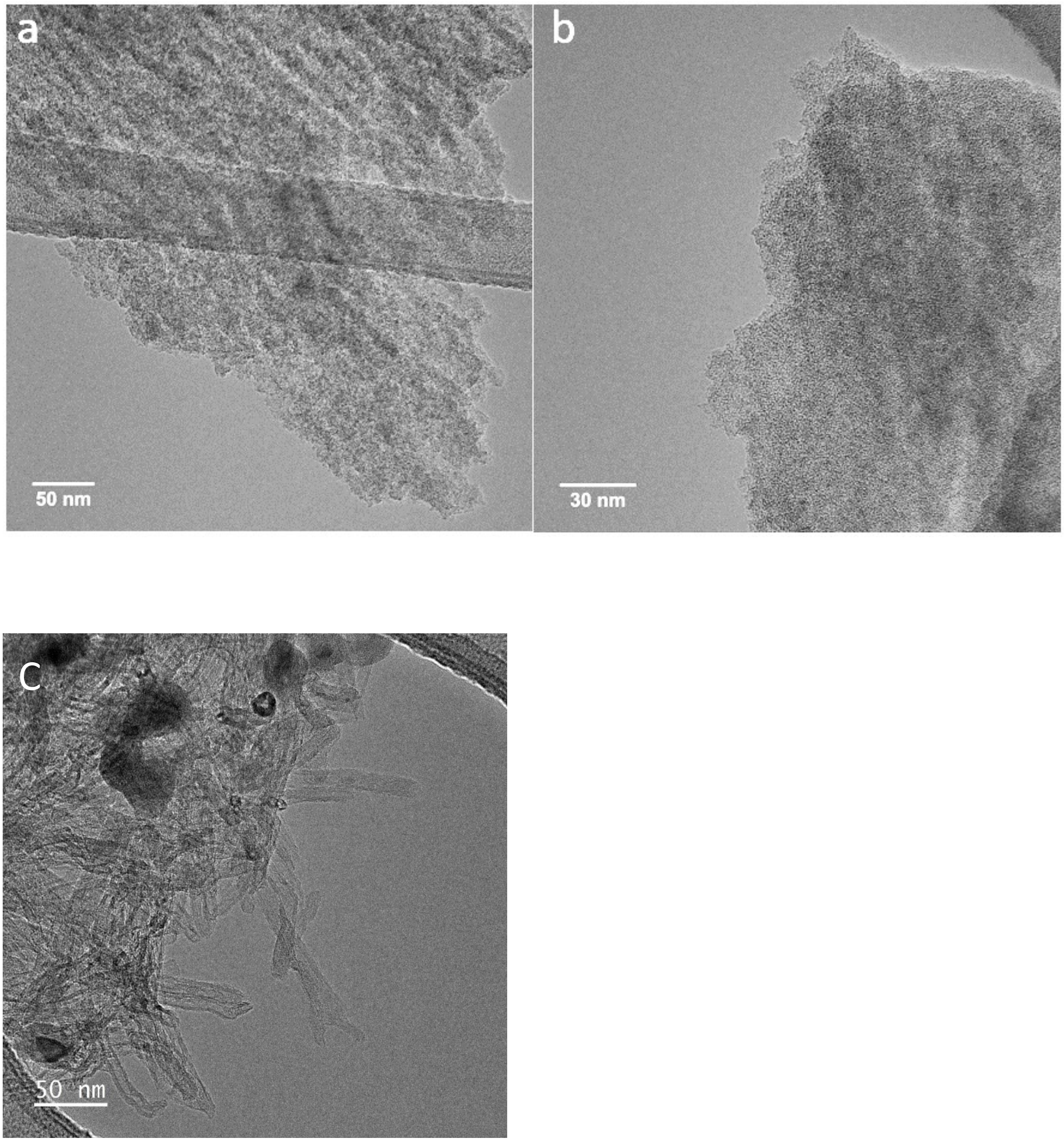

Figure S9 TEM images of fresh MoO_x/AC (a, b) and MoOx/CNF (c). The horizontal stripe is the TEM grid (lacey carbon).

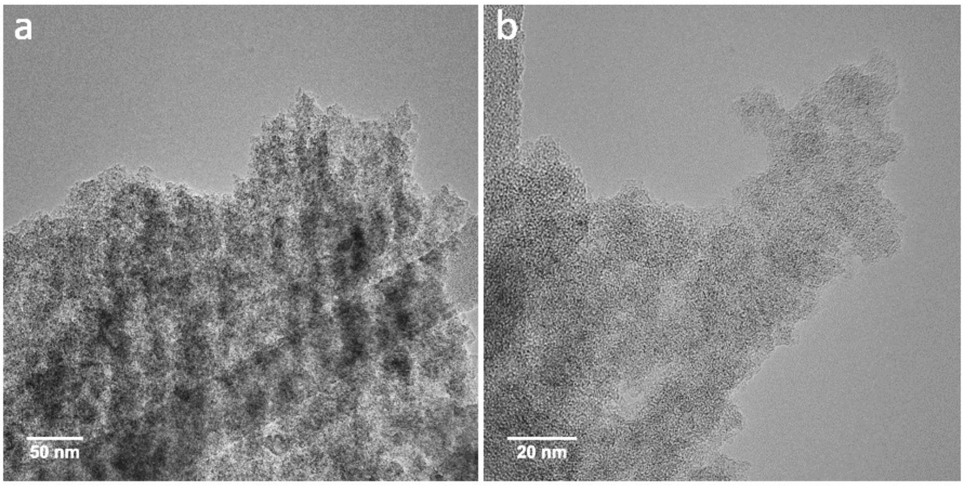

Figure S10 TEM images of fresh WO_x/AC.

Table S1 Physical properties of Mo-based carbon materials and their adsorption capacities.

Catalysts	SSA (m^2/g)	V_p (cm^3/g)	Sulfur adsorption capacity (μmol/g)	Sulfur adsorption per surface area (μmol/m^2)	Sulfate adsorbed in hydrothermal experiment (μmol)
AC	1539	1.3	6.0	0.0039	0.258
CNF	330	4.2	0.8	0.0024	0.034
MoO_x/AC	1036	0.8	3.8	0.0037	0.163
MoO_x/CNF	225	2.1	0.5	0.0022	0.021

SSA = specific surface area; V_p = total pore volume.

Table S2 Properties of materials tested as potential catalysts for hydrothermal ODS

Materials used catalysts	SSA (m^2/g)	Particle diameter (µm)	Composition
MoO_2	4	<0.1	99 % MoO_2
MoO_3	8	<0.1	99.5 % MoO_3
WO_3	-	<0.1	99.9 % WO_3
AC	1539	250-500	-
CNF	330	250-500	-
MoO_x/CNF	225	250-500	13 % Mo
WO_x/CNF	195	250-500	21 % W
MoO_x/AC	1036	250-500	13 % Mo
WO_x/AC	884	250-500	21 % W

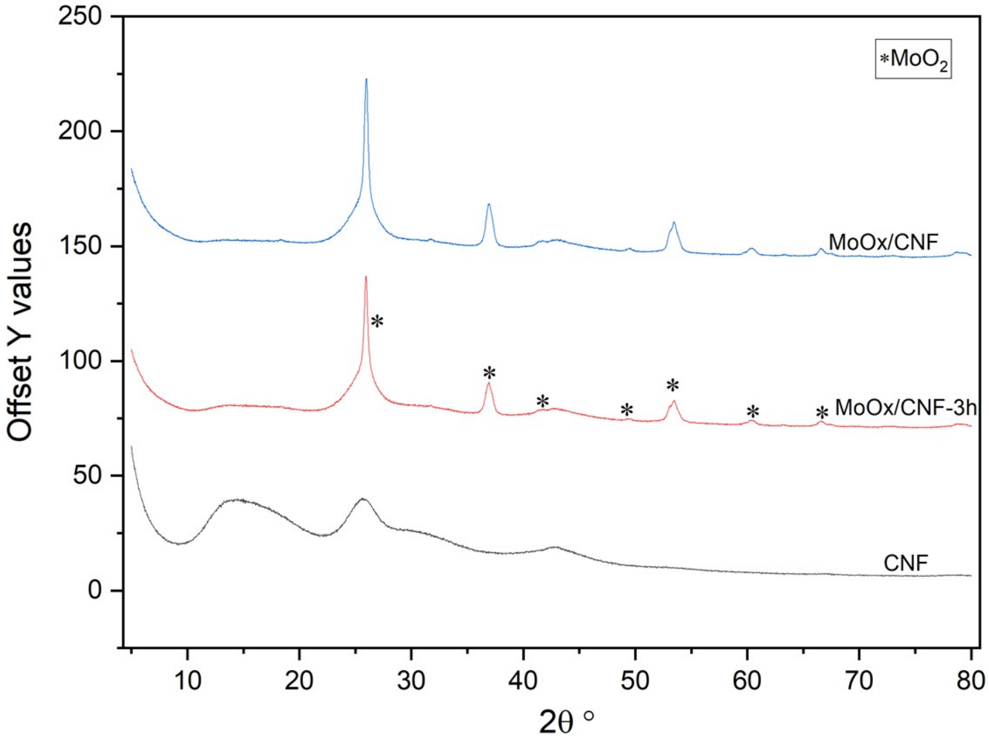

Figure S11 XRD patterns of MoOx/CNF with different calcination time.

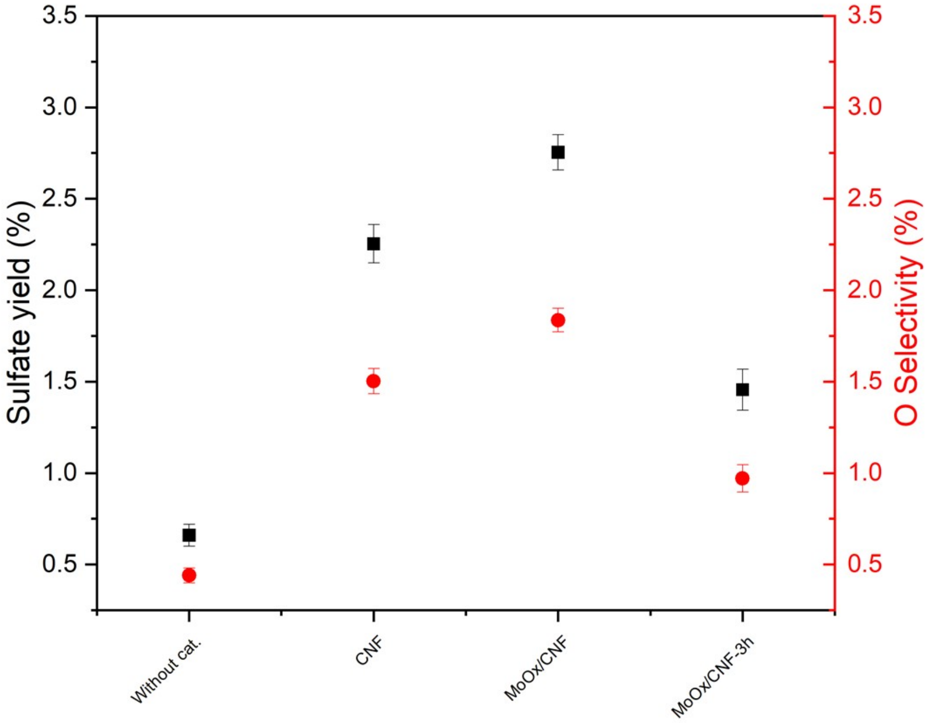

Figure S12 Influence of shorter calcination time of MoO_x/CNF on sulfate yield and oxygen selectivity (results from experiment 1, 18, 20 and 27).

Table S3 Micropore properties of materials tested as potential catalysts for hydrothermal ODS.

Support	SSA_{total} (m^2/g)	SSA_{micro} (m^2/g)	V_{total} (cm^3/g)	V_{micro} (cm^3/g)
AC	1539	1437	1.3	1.05
CNF	330	5	4.2	0

The micropore volume V_{micro} was determined by the t-method developed by Lippens and de Boer. The same calculation applies for the external surface area SSA_{micro}, defining the sample surface area without the micropore contribution. Pore size distributions were generated from the Barrett-Joyner-Halenda (BJH) model.

Figure S13 Pore size distributions (differential volume dV as a function of pore radius r) of the fresh AC and CNF.

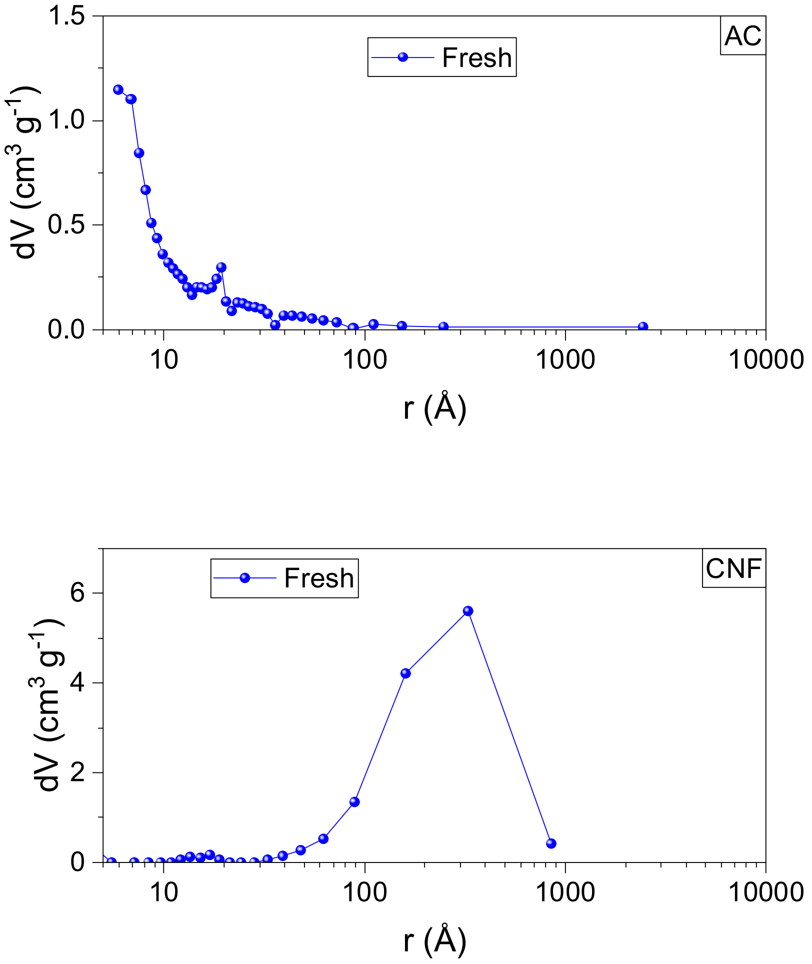

Supplement: SE-010-D5SE01500F-s001 [file SE-010-D5SE01500F-s001.pdf]
